# Isolation, Characterization, and Proteomic Analysis of Crude and Purified Extracellular Vesicles Extracted from *Fusarium oxysporum* f. sp. *cubense*

**DOI:** 10.3390/plants13243534

**Published:** 2024-12-18

**Authors:** Mudassar Ahmad, Yushan Liu, Shiyi Huang, Yile Huo, Ganjun Yi, Chongfei Liu, Wajeeha Jamil, Xiaofang Yang, Wei Zhang, Yuqing Li, Dandan Xiang, Huang Huoqing, Siwen Liu, Wei Wang, Chunyu Li

**Affiliations:** 1 Key Laboratory of South Subtropical Fruit Biology and Genetic Research Utilization, Ministry of Agriculture and Rural Affairs, Guangdong Provincial Key Laboratory of Science and Technology Research on Fruit Tree, Institute of Fruit Tree Research, Guangdong Academy of Agricultural Sciences, Guangzhou 510640, China; 2 Department of Pomology, College of Horticulture, China Agricultural University, Beijing 100193, China; 3 Institute of Tropical Bioscience and Biotechnology, Chinese Academy of Tropical Agricultural Sciences, Haikou 571101, China

**Keywords:** extracellular vesicles (EVs), *Fusarium oxysporum* f. sp. *cubense*, banana, gradient purification, proteomics

## Abstract

Extracellular vesicles (EVs) produced by *Fusarium oxysporum* f. sp. *cubense* (*Foc*) play vital roles in plant–pathogen interactions; however, the isolation of purified *Foc* TR4-EVs and their pathogenicity and proteomic profiles are not well studied. This study aims to isolate and characterize purified *Foc* TR4-EVs and compare their pathogenic effects and protein profiles with crude TR4-EVs. *Foc* TR4-EVs were isolated using ultracentrifugation and purified by iodixanol gradient centrifugation. After characterization and evaluation of the pathogenicity effects on banana leaves, LC-MS/MS was performed to conduct the proteomics assay. Results indicated that Fraction 2 EVs exhibited clearer spherical structures (TEM), excessive abundance (1.70 × 10^9^ particles/mL), greater intensity (400 a.u), mean size (154.5 nm), moderate protein content (333.16 ng/µL), and protein profile (25–77 kDa), which were superior to Fractions 1, 3, and crude EVs. Crude EVs displayed significant background interference with EV structures (TEM), highest abundance (2.11 × 10^9^ particles/mL), lower intensity (7.0 a.u), higher protein content (528.33 ng/µL), and higher molecular weight proteins (55–70 kDa) compared to gradient EVs. A non-significant biocontrol effect of Foc-EVs on the growth of TR4 spores was observed. Pathogenicity assays revealed that crude EVs caused the largest (2.805 cm^2^), while Fraction 2 (1.386 cm^2^) and Fraction 3 (1.255 cm^2^) resulted in moderate lesions on banana leaves. Proteomic analysis identified 807 unique proteins in Fraction 2, enriched in pathways related to EV trafficking and signaling. In comparison, crude EVs contained 179 unique non-EV proteins related to metabolism and secondary metabolites, indicating that non-EV proteins of crude EVs also influence the pathogenicity observed in banana leaves. This study emphasizes the importance of EV purification, with Fraction 2 being a critical focus for future research on *Foc* EV pathogenicity.

## 1. Introduction

*Fusarium oxysporum* f. sp. *cubense* (*Foc*) is a notorious polyphyletic soil fungus pathogen responsible for Panama disease in banana plants [1]. As the causal pathogen of this disease, *Foc* penetrates the plant’s vascular system, leading to wilting, chlorosis, and eventual plant death [2]. *Foc* pathogenicity leads to significant agricultural and economic losses globally that affect banana productivity, but this depends on the *Foc* race and the host’s susceptibility [3]. Based on the different host plant susceptibilities, *Foc* is classified into three distinct physiological races, including *Foc* races 1, 2, and 4 [2]. Among these, the *Foc* tropical race 4 (*Foc* TR4) is more phytotoxic and can infect various hosts, including the ‘Cavendish,’ a resistant cultivar to *Foc* races 1 and 2 [4]. The broad host range and virulence of *Foc* TR4 underscore the complexity of the pathogen’s interactions with banana plants. The pathogen has devastated banana plantations worldwide, and its spread to new geographic areas continues to exacerbate concerns for global food security and the banana trade [1]. This highlights the urgent need for in-depth research into its virulence mechanisms and the development of effective control strategies.

Among the various pathogenic mechanisms used by *Foc* TR4, the role of extracellular vesicles (EVs) in fungal pathogenicity has recently gained attention due to their capacity to facilitate cross-species interactions and deliver virulence factors to host cells [5]. EVs are nanoscale (30–2000 nm) round-shaped lipid bilayer particles secreted by prokaryotic and eukaryotic cells. They facilitate the virulence of pathogens by delivering encapsulated molecular cargo, including proteins, RNA, and other biomolecules from their lipid bilayer to host plant cells [6]. For instance, EVs can carry virulence factors across cellular boundaries, allowing fungi to compromise the plant’s immune response and exacerbate the effects of the infection. Plants, in turn, have evolved defense mechanisms, including trans-kingdom RNA interference, to counteract the pathogenic effects of these vesicles [7].

The field of EV research is expanding in mammalian systems, but there is an increase in EV research in the context of plant pathogens [8]. EVs have been identified from some filamentous plant-pathogenic fungi. To date, plant-fungal EVs have been studied in *Colletotrichum higginsianum*, anthracnose pathogen [9], *Fusarium oxysporum* f. sp. *vasinfectum* (Fov), cotton pathogen [10], *Ustilago maydis*, corn smut fungus [11], *Fusarium graminearum*, head blight fungus [12], *Penicillium digitatum* post-harvest rot pathogen [13], *Zymoseptoria tritici*, wheat pathogen [14], and *Blumeria hordei*, powdery mildew fungus [15]. Furthermore, injecting Fov EVs into the cotton leaves induced a phytotoxic response, suggesting Fov-EVs play a role in cotton infection, while proteomics revealed functional proteins related to cell wall degradation, secondary toxin metabolism, and protein degradation in Fov-EVs [10]. Fungal EVs export many virulence factors in a concentrated form as “virulence bags”, which can interact with host cells and promote infection [5]. A proteomics assay is necessary to understand these interactions and determine the proteins associated with these vesicles [16]. Proteins comprise the most significant EV cargo component and the key mediators of fungal virulence [16,17]. EVs isolated from the fungus *Z. tritici* and *F. graminearum* revealed several putative virulence-associated proteins that may cause infection, but some have no predicted secretion signal peptides [12,14]. Plant cells primarily internalize fungal EVs through endocytosis, which results in necrosis [6]. However, some researchers used EVs without gradient purification, such as crude EVs, to assess the pathogenicity [14]. These crude EVs may also have non-EV proteins and cellular debris, making it difficult to determine the specific contributions of EVs in pathogenesis [18,19].

EV isolation is the base of EV research, and different methods exist for EV isolation, like size-exclusion chromatography, filtration, differential centrifugation, and some precipitation-based kits [20]. Among these, differential centrifugation is widely regarded as the gold standard due to its capability to isolate EVs based on size and density, leading to high-purity EV samples [21]. Recent advancements in EV isolation techniques, particularly density gradient ultracentrifugation, have emerged as a critical criterion for EV purification [18]. During gradient centrifugation, a density gradient is established using a solution like sucrose or iodixanol, and the sample is subjected to high-speed centrifugation. As the centrifugation progresses, EVs migrate to their specific positions within the gradient according to their densities, resulting in distinct fractions [19]. This method facilitates the separation of EVs based on their buoyant density, yielding fractions with varying degrees of EV purity [22]. Further, techniques such as transmission electron microscopy (TEM) for EV morphology, the BCA assay for protein quantification, SDS-PAGE for protein profile analysis, and nanoparticle tracking analysis (NTA) to assess EV size distribution are beneficial to characterize these isolated EVs when specific biomarkers are unavailable [23].

Bananas, the world’s most important fruit in production and trade and among the world’s top 10 staple foods, are seriously vulnerable to filamentous fungi, such as *Foc* [24,25]. As the research into fungal EVs expands, significant questions remain regarding their isolation, characterization, phytotoxic functions, distinguishing fungal and host EVs, and identifying “hybrid” EVs from infected host cells without specific biomarkers [26]. Gradient purification techniques help overcome these obstacles by separating EVs on their buoyant density and improving the purity and yield of isolated EVs [21]. These two factors, purity and yield, are the two essential considerations for an EV isolation technique [22]. Despite the increasing recognition of EVs and their involvement to plant–pathogen interactions, the characterization and contribution of *Foc* TR4 EVs to disease progression are still underexplored. In our early study on *Foc* TR4-EVs, we aim to compare the differences in yield and purity between the crude EVs and those isolated in different fractions following gradient separation and to compare their pathogenic effects on banana leaves. Further, proteomic profiles of crude and purified EVs explore the differences in protein composition that may contribute to their virulence. By examining their physical properties, protein profiles, and pathogenic effects, we aim to clarify the role of purified EVs in *Foc* pathogenesis and emphasize the importance of EV purification in fungal virulence studies.

## 2. Materials and Methods

### 2.1. Foc TR4 Culture Growth

*Fusarium oxysporum* f. sp. *cubense* TR4 strain II5 (NRRL#54006), known as VCG 01213, was used in this study [27]. This strain was maintained on half-strength potato dextrose agar (½ PDA) at 28 °C. To prepare the *Foc* culture growth, spores were collected from the surface of ½ PDA and inoculated into sterile, half-strength 50 mL potato dextrose broth (½ PDB) and shaken at 28 °C, 180 rpm for 4–5 days as previously described [27].

### 2.2. Isolation and Gradient Purification of Foc TR4 EVs from Culture Media

To isolate *Foc* TR4 EVs, the post-incubated 50 mL spent medium was separated from the mycelia by filtering the *Foc* culture through four layers of sterile Miracloth. This cell suspension obtained in vivo was sequentially centrifuged to remove fungal spores and cell debris at 6500× *g* for 15 min, followed by 15,000× *g* for 30 min at 4 °C. The supernatant was filtered through 0.22 µm-pore syringe filters (Millipore, Burlington, MA, USA) to eliminate possible cellular debris and obtain a transparent conditioned medium (Figure S1). These centrifugation and filtration steps help remove large particles of cellular debris and remove contaminations. Firstly, we pelleted crude EVs from the growth cultures by ultracentrifugation for 2 h at 120,000× *g* at 4 °C in polypropylene quick seal 38.5 mL centrifuge tubes (Beckman Coulter, Brea, CA, USA, 342414) using a Type 70 Ti rotor and an Optima XE-90 ultracentrifuge machine (Beckman Coulter). Ultracentrifugation was repeated to wash the pellet with 1× PBS to obtain crude EVs to remove residual contaminants. These crude EVs were purified with an OptiPrep density gradient medium (OptiPrep; Sigma-Aldrich, St. Louis, MO, USA) using 40%, 30%, 15%, and 1% (*v*/*v*) iodixanol layers. To do this, crude EV pellets were resuspended in 1 mL of 0.1 M potassium acetate (pH 7.5) and mixed with 2 mL 60% OptiPrep to create a 40% layer. The 30% and 15% layers were made by diluting a 60% OptiPrep stock solution in 10× KoAC (5.39 g potassium acetate, 10 mL Hepes (1 M), 2.5 mL magnesium acetate tetrahydrate (0.5 M), and adding ddH_2_O up to 50 mL). The remaining 1% layer is a ~50 µL thin layer of 1× KoAC used to fill the tube up to the neck. The gradient was formed by sequentially adding each 1 mL layer to a 13 × 64 mm quickseal centrifuge tube (Beckman Coulter, Inc. 344619), starting with the 60% gradient with crude EVs (40%) and ending with a 1% thin layer. The gradient was centrifuged for 3 h at 120,000× *g* and 4 °C using a Type 100 Ti rotor and Optima XE-90 ultracentrifuge instrument (Beckman Coulter). The following four 1 mL fractions were collected for further ultracentrifugation to obtain the pellet of each fraction. Samples were added to 13 × 32 mm quick seal small ultracentrifugation tubes (Beckman Coulter, Inc., 349621) and brought up to a volume of 3.5 mL using cold, 1× PBS. Samples were centrifuged for 30 min at 120,000× *g* and 4 °C using a Type 100 Ti rotor to obtain the pellets of each fraction. The supernatant was decanted entirely, and the pellet of each fraction was resuspended in 20–30 µL 1× PBS pH 7.5 for further experiments. The final washing and resuspension in PBS ensured that only the purified EVs that were free of contaminants remained.

### 2.3. Transmission Electron Microscopy (TEM)

Foc-EVs were observed under TEM following the method [10]. Briefly, EVs (10 µL, conc. 1 × 10^8^ particles/mL) were applied onto carbon-coated copper grids and exposed to glow discharge. Further, grids were washed with ultrapure water, stained with 10% uranyl acetate, and dried overnight. Imaging was performed using a HITACHI Transmission Electron Microscope (HT7700, Hitachi, Tokyo, Japan).

### 2.4. Nanoparticle Tracking Analysis (NTA)

Particle size, concentration, and intensity of purified EVs were measured using a Nanosight NS300 (Malvern Panalytical Ltd., Malvern, UK) according to the manufacturer’s user handbook (MAN0541-01-EN-00, 2017), and the analysis was conducted using NanoSight Software NTA3.3.301 (Malvern Panalytical Ltd., Malvern, UK). Samples were diluted with sterile 1× PBS and injected using a syringe pump to adjust their concentration by monitoring a particles/frame rate of approximately 60 (ranging from 60 to 100 particles/frame). Five consecutive 60 s standard measurements were performed. EVs were identified using a 488 nm laser (blue) and a scientific CMOS camera to obtain information, including the mean size, mode (the most observed EV size in the population), intensity, and the number of particles/mL.

### 2.5. Bicinchoninic Acid Assay (BCA)

The total protein concentration of Foc-EVs was determined using the Modified BCA Protein assay kit (Ref C503051-0500 Songon Biotech, Shanghai, China) following the manufacturing instructions. Briefly, the BCA Reagent A and Reagent B were mixed in a 50:1 ratio to prepare a working reagent. Firstly, a series of BSA standards with concentrations 0, 25, 125, 250, 500, 750, and 1000 ng/µL were added into a 96-well microplate, and then three replicates of 10 µL of EV samples mixed with 200 µL BCA working reagent were added and incubated at 37 °C for 30 min. The absorbance was measured at 562 nm using a Tecan Spark multimode microplate reader, and the standard curve was made to determine the protein concentration of the fungal EV samples based on their absorbance values.

### 2.6. Sodium Dodecyl Sulfate-Polyacrylamide Gel Electrophoresis (SDS-PAGE)

SDS-PAGE assay was performed to separate Foc-EV proteins according to their molecular weight. Briefly, 10 µL of Foc-EV samples were mixed with an equal volume of 2× SDS buffer and incubated at 98 °C for 10 min to disrupt EV membranes and denature their proteins. After loading the samples and protein ladder on 10% SDS-PAGE gel (SurePAGE^TM^, GenScript, Rijswijk, The Netherlands), electrophoresis was performed at a voltage of 80 V for two hours. The gel was dyed with Coomassie Brilliant Blue (Beyotime, Shanghai, China) and then destained with water to remove excess dye, and protein bands were observed against a white light background.

### 2.7. Leaf Phytotoxicity Assay

Healthy, pristine banana seedlings at three to four leave stages of the Brazilian banana cultivar, a premium banana cultivar with high-quality flavor [27], were chosen. The leaves were carefully detached and cleaned adequately with sterile water and air-dried. Previous studies have also utilized detached leaves for EV research and *Foc* infection because they provide a simplified, controlled system with a precise observation of localized responses for studying the direct interaction between pathogens and host tissue [10,27]. *Foc* TR4 EVs concentration was adjusted at 1 × 10^8^ particles/mL. Four incisions were made on the abaxial surface of each leaf using a sterile scalpel or pinpricked, positioned approximately equidistant from the midrib and major veins. At each incision site, 20 µL of crude or gradient Foc-EV, along with 1× PBS (control), was applied at each leaf, and a piece of sterile soaking paper was applied to cover each incision. The leaves were subjected to vacuum pressure for 5 min and placed in plastic boxes lined with wet tissue papers to preserve humidity. The boxes were placed at 28 °C under dark conditions for three days to examine for any signs of phytotoxicity, such as discoloration, necrosis, or lesion formation, and then photographed. Necrosis formation on banana leaves was measured using ImageJ v1.8.0 software. Three independent experiments were performed to verify the results, and then samples were collected for further studies.

Trypan blue was used to examine cell death. Briefly, after incubation, the leaves were stained overnight with 0.02% trypan blue (volumetric ratio, 1:1:1:1:8::lactic acid:phenol:glycerol:water:ethanol) at room temperature. The leaves were destained first immersed in 75% and then 95% ethanol solution until they attained a uniform, transparent color. Leaves were then photographed using a Nikon camera.

### 2.8. RNA Extraction and qRT-PCR

Total RNA extraction and qRT-PCR were performed using the method described before [28]. In brief, an RNA out kit (Tiandz, Beijing, China) was used to extract RNA from the infected area of banana leaves, and AMV Reverse Transcriptase (Takara, Osaka, Japan) was used to synthesize cDNA, and finally, quantitative real-time reverse transcriptase PCR (qRT-PCR) was performed using StepOne real-time PCR system (Applied Biosystems, Carlsbad, CA, USA). *MaActin* was used as the reference gene for normalization, and the expression of the previously reported banana immune marker genes, including MaPR1a, MaPR2, MaWKRY12, MaPI1, and MaHIN1, were analyzed [27]. Three biological replicates were used in this experiment utilizing the primers listed in Table S1.

### 2.9. Biocontrol Effects of EVs on Spores of Foc TR4

To evaluate the biocontrol effects of *Foc* TR4 EVs on its spores, we followed a method adapted from previous studies [29]. Firstly, the *Foc* TR4 culture (50 mL) was incubated on PDB, and the post-incubated spent medium was separated from the mycelia by filtering the culture through four layers of sterile Mira cloth. The filtered medium was then centrifuged at 6500× *g* for 10 min to collect the spores. The supernatant was discarded, and the spore pellet was resuspended in 20 mL of sterile distilled water (ddH_2_O). The spore suspension was adjusted to a concentration of 1 × 10^5^ spores/mL using the hemocytometer. For the treatment, 20 µL of *Foc* TR4 EVs (1 × 10⁸ particles/mL) was mixed with 20 µL of the TR4 spore suspension. For the PBS effect, 20 µL of 1× PBS was mixed with 20 µL of the spore suspension, as the EV pellet was dissolved in 1× PBS. The control involved mixing 20 µL of spores with 20 µL of ddH_2_O. 10 µL of each resulting mixture was applied to the center of a PDA plate. Three plates of each inoculation were incubated at 28 °C for six days, and the colony sizes were measured at 2, 4, and 6 days using ImageJ v1.8.0 software.

### 2.10. LC-MS/MS

Samples were analyzed on a Bruker timsTOF Pro 2 mass spectrometer (Bruker, Bremen, Germany) coupled with UltiMate 3000 RSLCnano (Thermo Fisher Scientific, Waltham, MA, USA). After protein extraction and preparation from EVs, dried peptide samples were dissolved in 20 µL of 0.1% formic acid in water, and 1 µL was loaded onto the Acclaim™ PepMap™ 100 C18 trap column (5 mm × 1 mm; particle size, 5 µm; pore size, 100 Å; ThermoFisher Scientific, Waltham, MA, USA), then separated on the Aurora™ ULTIMATE C18 analytical column (25 cm × 75 µm; particle size, 1.7 µm; pore size, 120 Å; IonOpticks, Fitzroy, Australia) with a gradient of 5–35% mobile phase B (acetonitrile and 0.1% formic acid) at a flow rate of 300 nL/min for 65 min. The gradient started at 2% of B in 2 min, and the composition of solvent B was increased to 5% in 0.1 min. It was then increased from 5% to 35% in 44.9 min, from 35% to 90% for 4 min, and then washed at 90% for 5 min. % B was then decreased back to 2% in 0.1 min and was held for 4.9 min. MS and MSMS spectra were acquired at a mass scan range of 100–1700 *m*/*z* and an ion mobility scan range of 0.6–1.6 V·s/cm^2^. The dual TIMS setup allows the system to operate at 100% duty cycle, and the ramp and accumulation time are set at 100 ms. The MS/MS spectra were acquired using library-free DIA-PASEF (Data independent acquisition-Parallel Accumulation-Serial Fragmentation) mode, in which the total cycle time is 1.8 s (isolation window 25 *m*/*z*) and the collision energy is from 59 eV at 1.3 Vs cm^−2^ to 20 eV at 0.85 Vs cm^−2^.

### 2.11. Proteome Identification and Quantification

Database search and label-free quantification were performed using the SpectronautTM ^17^ search engine (Biognosys Inc., Cambridge, MA, USA) in the library-free (directDIA) workflow. The database was downloaded from UniProt. Taxonomy ID 1089451 was used for *Foc* TR4. The protein identification search was performed using the following parameters set: (1) fixed modification: cysteine carbamidomethylation (+57.021 Da); (2) variable modification: methionine oxidation (+15.995 Da) and acetylation (+42.011 Da) at the protein N terminus; (3) trypsin was used for digestion with two missed cleavages allowed; (4) Only PSM, peptides, and protein groups with false discovery rate (FDR) < 1% and protein with one or more unique peptides were filtered out. Label-free quantification was performed based on the identification search result with an FDR threshold of 1% at the MS2 level using the area of extracted chromatogram traces. The automatic-based normalization strategy was selected.

### 2.12. Data Analysis

Proteomic data analysis was conducted to characterize and identify total and differentially enriched proteins (DEPs) in Foc-EVs. Proteomic samples were analyzed in biological triplicate for each experimental condition. Total protein Venn diagrams of Foc-EVs were generated using TBtools [30], and then both groups were subjected to Gene Ontology (GO) and Kyoto Encyclopedia of Genes and Genomes (KEGG) enrichment analyses. Differential expression proteins were performed using DESeq2 v1.35.0 [31] following read count normalization. Proteins were classified as differentially enriched if they had a log2 fold change ≥ 1 and an adjusted *p*-value (q-value) ≤ 0.05.

## 3. Results

### 3.1. Isolation and Characterization of TR4-EVs by TEM and NTA

Extracellular vesicles (EVs) from *Foc* TR4 strain II5 were initially extracted using ultracentrifugation methods (Figure S1). After crude extraction, the EVs underwent further purification through iodixanol density gradient centrifugation, where they were separated into three distinct layers based on 15%, 30%, and 40% density. The EVs collected before gradient purification were termed “crude EVs”, while those isolated from the gradient layers were designated as Fraction 1 (F1, 15%), Fraction 2 (F2, 30%), and Fraction 3 (F3, 40%) (Figure S1). These gradient-purified fractions were selected for further characterization to identify the most abundant EV fraction of *Foc* TR4.

**Figure 1:**
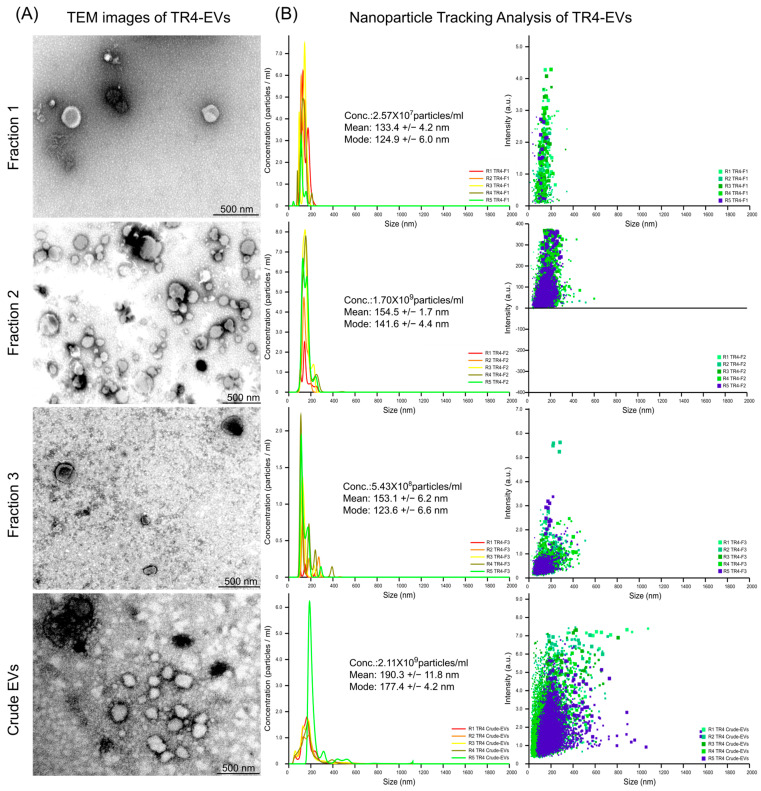
Characterization of TR4-EVs by TEM and NTA. (**A**) Transmission electron microscopy (TEM) images of TR4-EVs illustrate the morphology and purity of different EV fractions (Fraction 1, Fraction 2, Fraction 3) and the crude EV preparation. The scale bars represent 500 nm. (**B**) Nanoparticle tracking analysis (NTA) quantifies the concentration, size distribution, and intensity of particles within the same fractions and crude EVs, providing complementary insights into EV characteristics.

To understand the morphology and purity of *Foc* TR4-EVs, we first performed TEM across various fractions (Fraction 1, Fraction 2, and Fraction 3) and the crude EV preparation (Figure 1A). Fraction 1 contains more clean, well-defined, round-shaped EVs, though it has a relatively low number compared to other fractions. Fraction 2 contains the highest number of spherical-shaped EVs with distinct structures, making it the most refined and abundant fraction than others. In contrast, Fraction 3 contains fewer EVs with increased background particles, reducing overall purity. Similarly, the crude EV preparation displays a mixture of EVs and a substantial number of cellular debris or non-EV particles, indicating a lower refinement level than the purified fractions (Figure 1A).

Subsequently, NTA was conducted to quantify the particle concentration, size distribution, intensity, and overall abundance of EVs to compare gradient and crude EVs, providing complementary insights into their physical characteristics (Figure 1B). Fraction 1 has a particle concentration of 2.57 × 10^7^ particles/mL, with a mean size of 133.4 nm and a mode size of 124.9 nm. Fraction 2, in contrast, shows the highest particle concentration at 1.70 × 10^9^ particles/mL, with a mean size of 154.5 nm and a mode size of 141.6 nm, along with the highest intensity, indicating a significant presence of EVs. Fraction 3 has a concentration of 5.43 × 10^8^ particles/mL, with a mean size of 153.1 nm, but lower concentration and intensity than Fraction 2. The crude EV sample, with a concentration of 2.11 × 10^9^ particles/mL and a larger mean size of 190.3 nm, indicates a mix of EVs and non-EV particles. Fraction 2 shows the highest intensity (up to 400 a.u.) compared to other fractions (up to 5 a.u) and crude EVs (up to 7 a.u.). This higher intensity of Fraction 2 likely reflects a greater degree of homogeneity in the size, shape, and composition of the vesicles, which contributes to more efficient light scattering during NTA (Figure 1B). Hence, Fraction 2 shows the round shape with the highest EV concentration, purity, and intensity, indicating a well-defined and abundant EV population of *Foc* TR4.

### 3.2. Protein Concentration and Profile of TR4-EVs by BCA and SDS-PAGE

**Figure 2:**
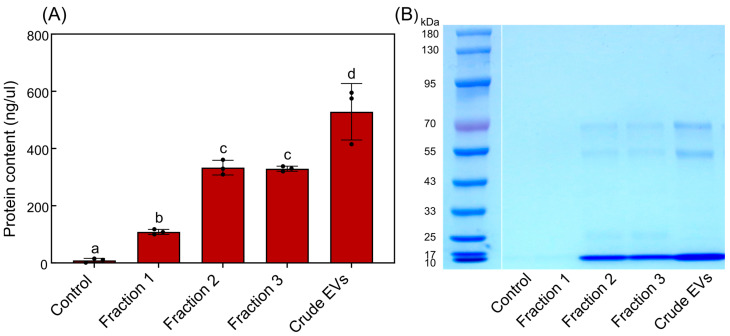
BCA and SDS-PAGE analysis of TR4-EVs. (**A**) BCA assay results indicate the protein concentrations of each fraction and crude EVs compared with control (1× PBS). Means followed by the same letter(s) are not significantly different at the 5% level analyzed by Duncan’s multiple range test. (**B**) SDS-PAGE analysis displays the protein bands of each fraction and crude EVs. A molecular weight ladder included for reference ranges from 10 kDa to 180 kDa for accurate identification of band sizes. Three independent experiments were conducted to confirm the results.

The BCA assay was performed to compare the protein content of the different fractions of TR4-EVs with its crude EVs (Figure 2A). The results reveal varying protein concentrations across the fractions. Fraction 1 has a relatively low protein concentration of 108.5 ng/µL. In contrast, Fraction 2 exhibits a moderate protein content at 333.16 ng/µL, indicating a significant enrichment of EVs compared to the other fractions. Fraction 3 has a similar protein level of 325 ng/µL as Fraction 2 without significant difference. Interestingly, the crude EV preparation has a higher protein concentration of 528.33 ng/µL, likely due to a more significant total protein content that includes EVs and non-EV outer proteins (Figure 2A). The ultra-high protein concentrations and appearance in TEM indicated that some aggregate proteins or cellular debris are also present in crude EVs.

Further, SDS-PAGE analysis was conducted to examine the distinct protein bands of each fraction and crude EVs of *Foc* TR4-EVs (Figure 2B). To validate the protein concentration differences observed in the BCA assay, we loaded equal volumes (10 µL) of Foc-EV samples for SDS-PAGE analysis. This approach allowed us to compare protein band intensities based on their molecular weight and validate protein abundances across the fractions. Our analysis shows distinct band intensities for each sample in comparison to the control. The bands appear at lower intensity in Fraction 1, mainly around the 25–45 kDa range, indicating negligible amounts of EVs. Fraction 2 is distinguished by the most prominent bands between 55–77 and 25–45 kDa, reflecting both higher and low molecular protein. Fraction 3 shows bands in the 55–77 kDa range, like Fraction 2, but with low intensity at 25–45 kDa. The crude EV sample displays a broad range of bands, with thick bands around 55–70 kDa and negligible bands at 25–45 kDa, revealing high molecular weight proteins are present in abundance (Figure 2B). It suggests that crude preparations may carry abundant non-EV or protein aggregates that co-purify with EVs during the isolation process. Overall, the BCA and SDS-PAGE results reinforce the findings from TEM and NTA, highlighting that Fraction 2 has the highest EV concentration and purity and presents significant protein content and a distinct protein profile from 25–77 kDa (Figure 1 and Figure 2).

### 3.3. Pathogenic Effects of Gradient and Crude EVs on Banana Leaves and Host Immune Response

**Figure 3:**
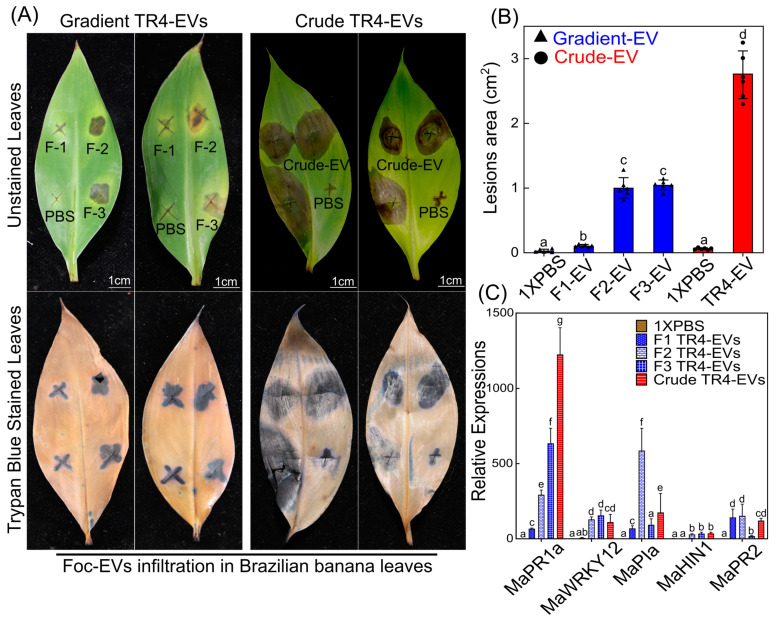
Pathogenic effects of Foc-TR4 fractions and crude EVs on banana leaves. (**A**) Photographic representation of banana leaves injected with PBS (control), F1 (Fraction 1), F2 (Fraction 2), F3 (Fraction 3), and EVs (crude EVs), with lesion areas assessed 4 days post-infiltration. Scale bar = 1 cm. (**B**) Quantification of lesion areas (mean ± SE) for each treatment group. Significant differences among treatments were determined using one-way ANOVA followed by Duncan’s multiple range test (*p* < 0.05). Different letters (a, b, c, d) indicate statistically significant differences. (**C**) Expression analysis of immune marker genes *PR1a*, *PR2*, *WKRY12*, *PI1*, and *HIN1* in banana leaves at the infiltration site, comparing immune responses to various EV treatments. Means followed by the same letter(s) are not significantly different at the 5% level, as analyzed by one-way ANOVA and Duncan’s multiple range test.

Gradient fractions and crude EVs of *Foc* TR4 were infiltrated on Brazilian banana leaves to evaluate the pathogenic effects of the different EV preparations (Figure 3A). We found that crude EVs caused the most significant leaf lesions, averaging 2.805 cm^2^ (Figure 3A,B). This widespread lesion area proposes that the higher proteins of crude EVs possibly enhance pathogenicity. In contrast, Fraction 2 and Fraction 3 produced moderate lesions, with areas of 1.386 cm^2^ and 1.255 cm^2^, respectively (Figure 3B). Fraction 2 has a higher EV content, while Fraction 3 contains more cellular debris or free protein and fewer EVs, suggesting that both components contribute to pathogenic effects in banana leaves. Fraction 1, with an average lesion area of 0.508 cm^2^, produced a response nearly identical to the PBS control, which showed no observable effect on the banana leaves. This minimal lesion formation indicates that Fraction 1 may contain a lower concentration of pathogenic factors, resulting in limited tissue damage (Figure 3A,B).

Additionally, the expressions of banana immune marker genes are upregulated at the infiltration sites, indicating significant immune activation in banana leaves treated with crude and gradient EVs (Figure 3C). We found that *MaPR1a*, a marker for systemic acquired resistance (SAR), *MaWRKY12*, and *MaPIa* were significantly expressed under all the treatments compared to the control. Fraction 2 and crude EVs highly induced *MaPIa* and *MaPR1a* expressions, respectively. These high expressions suggest that EVs may induce an immune response in banana leaves, resulting in the necrosis observed at the infiltration sites (Figure 3C). These findings underline that the host immune system may respond differently to EV purity and content in modulating pathogenicity.

### 3.4. Biocontrol Effect of TR4 Gradient and Crude EVs on Spore Germination

**Figure 4:**
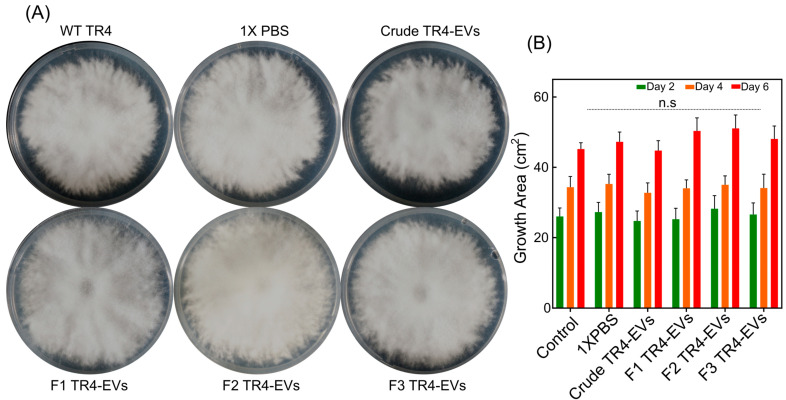
Biocontrol potential of purified fractions and crude EVs of *Foc* TR4 on spore germination. (**A**) Fresh EV samples (conc. 1 × 10^8^ particles/mL) are gently mixed with spores (1 × 10^5^ spores/mL) in PBS and inoculated in the center of the PDA medium plate for 6 days. WT and spores mixed with sterile 1× PBS are used as controls. (**B**) Bar graphs represent the colony growth area for *Foc* EVs. Colony size is measured using the ImageJ software, while the plate area is 63 cm^2^. Error bars representing the standard deviation (SD) of three biological replicates and ns indicate non-significant differences (Student’s *t*-test, *p* < 0.001).

To examine the possible biocontrol effects of gradient-purified and crude EVs on TR4 spores, we performed an in vitro experiment (Figure 4). In this experiment, TR4 spores (1 × 10^5^) were mixed with crude and each fraction EV (1 × 10^8^ particles/mL) and placed at the center of PDA plates. Three separate EV fractions (F1, F2, and F3) and crude EVs were compared with control and 1×PBS. Spore germination and colony sizes were measured on days 2, 4, and 6. After six days of incubation, quantitative analysis revealed that Fraction 1 and Fraction 2 showed a greater effect on the germination of TR4 spores that nearly covered the entire PDA plate than the control and 1× PBS treatments; however, the difference was not statistically significant (Figure 4B). Similarly, no significant difference in spore germination was observed on days 2 and 4, consistent with the results on day 6 (Figure 4B). All EV treatments had a non-significant impact on the spore germination of TR4 in comparison to 1×PBS and control groups. This suggests that EVs may have affected TR4 growth; however, the impact was not statistically significant under the PBD conditions (Figure 4).

### 3.5. Distinct Proteomic Profiles and Functional Enrichment in Fraction 2 and Crude EVs

**Figure 5:**
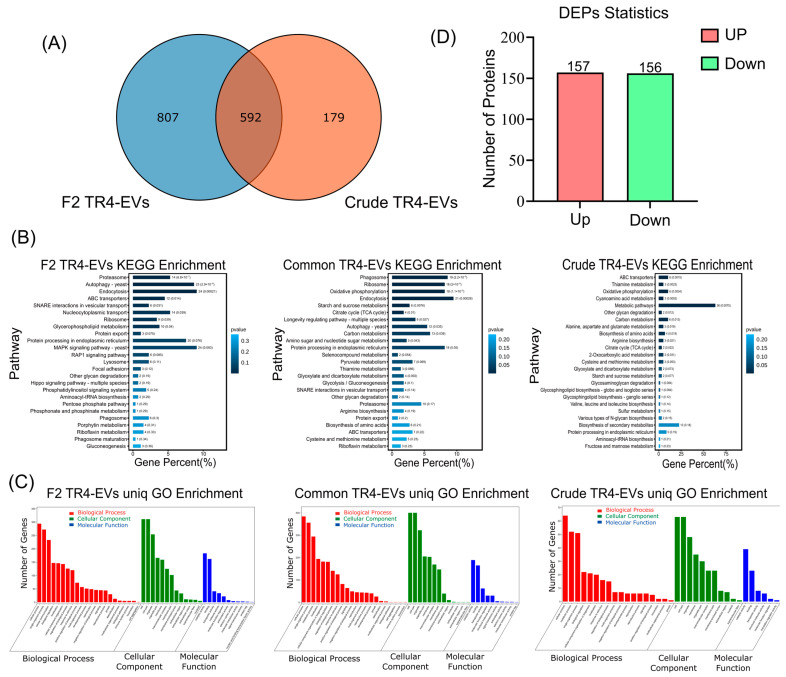
Proteomic comparison between Fraction 2 (F2) EVs and crude TR4 EVs. (**A**) Venn diagram displaying unique and shared protein counts of Fraction 2 and crude TR4 EVs (**B**) KEGG pathway enrichment analysis showing EV-specific and non-EV pathways of Fraction 2 and crude TR4 EVs, respectively. (**C**) GO enrichment analysis highlighting cellular compartment, biological process, and molecular function categories of Fraction 2 and crude TR4 EVs. (**D**) Differentially enriched proteins (DEPs) in F2 EVs compared to crude EVs, with a balanced number of up- and down-regulated proteins reflecting divergent functions between the preparations.

Because Fraction 2 (F2) carried the purified form of TR4-EVs, a proteomic analysis between F2 and crude TR4 EVs was performed to delineate the protein composition and functionality (Figure 5). We identified 1399 total proteins in Fraction 2 and 771 total proteins in crude EVs, respectively, and further, the Venn diagram illustrates 807 unique EV-related proteins in F2 EVs and 179 unique non-EV-related proteins in crude EVs, with 592 shared proteins of both types of EVs (Figure 5A).

Our KEGG analysis of F2 TR4-EVs revealed significant enrichment in pathways related to vesicular transport, including SNARE interactions, ABC transporters, autophagy, protein export, and endocytosis, all of which are critical for EV functionality in intercellular communication and cargo delivery (Figure 5B). Furthermore, pathways involving protein processing in the endoplasmic reticulum, proteasome, lysosome, and phagosome-related proteins were notably enriched in F2 EVs. The MAPK, RAP1, and phosphatidylinositol signaling pathways, crucial for EV signaling, are also abundant in F2 EVs (Figure 5B). Conversely, the unique protein of crude TR4-EVs indicated pathways enriched in metabolism and secondary metabolites, implying the existence of non-EV proteins. The presence of phagosomes, endocytosis, autophagy, SNARE interactions in vesicular transport, protein export, and proteins associated with ABC transporters in common proteins are evidence of EV presence in crude samples (Figure 5B). The unique gene names related to different KEGG pathways of *Foc* EVs are shown in Table 1. The differences indicate that F2 EVs have EV-specific functional pathways, whereas crude EVs also contain non-EV protein pathways.

The Gene Ontology (GO) enrichment also shows these differences (Figure 5C). Cellular compartments in F2 TR4-EVs included extracellular matrix, extracellular space, and vesicle membrane, consistent with EV-specific features. At the same time, crude TR4-EVs were enriched mainly for broader cell membranes and organelle components, indicating a mixture of intracellular components. Regarding biological activities, F2 TR4-EVs exhibited greater enrichment in cell adhesion, immune response modulation, and signal transduction, consistent with the functional characteristics anticipated in pure EVs. Crude EVs, however, included cellular metabolism and stimulus-response functions, further suggesting non-EV-related genes (Figure 5C).

Furthermore, the differentially enriched proteins (DEPs) analysis reveals a similar quantity of upregulated (157) and downregulated (158) proteins across F2 and crude EVs (Figure 5D). The even distribution of DEPs indicates that both preparations are functional but have different roles, with F2 EVs directed towards vesicular transport and signaling, while crude EVs exhibit non-vesicular, potentially cellular residue functions, underscoring the importance of the purification process on the functions of *Foc* EVs (Figure 5D).

## 4. Discussion

### 4.1. Ensuring EV Isolation by Gradient Purification Is Necessary

In the past decade, there has been a substantial increase in scientific publications focusing on the physiological and pathological roles of extracellular vesicles (EVs) [32]. These cell-released membranous structures play important roles in intercellular communication, but their small size and heterogeneous nature present significant challenges in isolating them in pure forms [23]. The International Society for Extracellular Vesicles (ISEV) introduced the Minimal Information for Studies of Extracellular Vesicles (MISEV) guidelines in 2014 to standardize EV research, later updated as MISEV2018 to incorporate new advancements. The recommendations of these guidelines emphasize the need for high-purity EV preparations since gradient centrifugation is crucial for acquiring pure EV samples and ensuring precise functional attributions by reducing contamination from other cellular components or protein aggregates in crude preparations [18].

Further, recent studies that isolated fungal EVs without gradient purification and also infiltration of these EVs showed necrotic effects on plants [13,14,33]. Crude preparations can be contaminated with free proteins, residual cellular components, co-isolated materials, and other non-EV entities that complicate functional attributions [23,34]. Similarly, proteomic analysis of without gradient EVs and soluble supernatants from *Z. tritici* (wheat fungus) revealed the co-occurrence of specific proteins, suggesting that it was due to the contamination [14]. This indicates that some fungal EV proteomes, which serve as sources for choosing biomarkers or virulence factors, likely also contain non-EV molecules [9]. These studies also highlight the necessity of including purification steps like density gradient centrifugation to remove non-vesicular components [13,14,18]. Therefore, gradient purification is essential to isolate EVs from other isolated materials to ensure that observed biological effects are truly vesicle-derived [35].

### 4.2. Gradient-Purified Fraction 2 Carries Superior Abundance, Size, and Purity of Foc TR4 EVs

In our study, we isolated the *Foc* TR4 EVs by gradient centrifugation (Figure S1) and compared the various fractions of the gradient with crude EVs. Firstly, the purity of EV samples observed by TEM indicates that crude EVs and Fraction 3 EVs contain more background interferences than Fractions 2 and 1, and among all, Fraction 2 carried the refined and dense EV of *Foc* TR4 (Figure 1A). Fraction 2 exhibits a particle concentration of 1.70 × 10^9^ particles/mL, while Fraction 1 is highly refined but contains a relatively lower dense 2.57 × 10^7^ particles/mL (Figure 1B). Previously, EVs isolated from *C. higginsianum* by density purification highlighted particle density in fractions three, five, and six of ~1.078, ~1.115, and ~1.173 g/mL, respectively, indicating high-density (HD) population in fractions five and six and low-density (LD) population in fraction three [9]. In contrast, crude EVs showed a significantly higher concentration of 2.11 × 10^9^ particles/mL than all fractions (Figure 1B). Similarly, after ultrafiltration/diafiltration, isolated crude EVs from hamster ovary showed the highest particle concentration (1.10 × 10^12^ p/mL) than all fractions, including Fraction 3 (2.39 × 10^11^ p/mL), Fraction 4 (3.08 × 10^10^ p/mL). and Fraction 5 (7.54 × 10^9^ p/mL) [36]. The highest particle indicates the presence of non-EV particles that negatively affect both the quality and the potential applications of these EVs [37]. Further, the mean size of Fraction 2 EV particles (154.5 nm) is similar to those from other filamentous fungi like *Fo vasinfectum* (155 nm) [10], *T. reesei* (144 nm) [38], and *T. interdigitale* (110 nm) [39] compared to crude EVs that showed large-size particles (190.3 nm). Comparable studies have also shown that gradient fractions provide superior purity and functionality relative to crude extracts, underscoring the need to use stringent purification techniques [40]. This superior abundance and size of Fraction 2 is an optimal candidate for future investigation and implementation of *Foc* TR4 EVs in disease management.

### 4.3. Fraction 2 Has Superior Protein Content and Unique Proteomic Profiles

It is well known that protein quality and functionality are essential in EV applications; for example, Kalluri and LeBleu (2020) highlight that EVs must have specific protein composition in order to function well in biological processes [35]. Our findings indicated that Fraction 2 exhibited a protein concentration of 333.16 ng/µL, above that of other fractions, but crude EVs recorded the greatest overall concentration at 528.33 ng/µL (Figure 2A). The lower protein content might appear in purified EVs due to removing cellular debris or protein aggregates. SDS-PAGE analysis indicated separate protein bands in Fraction 2 (25–77 kDa), while crude EVs exhibited a higher concentration of high molecular weight proteins (55–77 kDa) (Figure 2B). This Fraction 2 protein profile is similar to *Candida* (15–75 kDa) [41], *Cryptococcus neoformans* (25–80 kDa) [42], *Aspergillus flavus* (20–90 kDa) [43], and *Saccharomyces cerevisiae* (25–70 kDa) [44] fungi. The proteome analysis of *Escherichia coli* EV stains reveals alterations in protein abundance during purification, with the 60 kDa chaperonin GroEL being enriched in the crude input EVs but absent following purification via density gradient centrifugation (DGC), indicating that DGC effectively removes protein aggregates and cellular structures [45]. Our crude EVs had a broader, less distinct protein profile, suggesting the presence of contaminants that might impair their biological efficiency. The similar band pattern observed in Fraction 3 (25–77 kDa) may result from gradient separation, where most EVs are shifted from Fraction 3 to Fraction 2, leaving protein aggregates, cellular debris, and some EV particles behind. This might explain the overlapping protein weight ranges between Fraction 2, Fraction 3, and crude EVs, but with differing protein abundances (Figure 2B). The variations in protein composition highlight the benefits of using gradient centrifugation methods for obtaining purified EVs, which substantially surpassed crude extracts in true EV functional applications [21]. Thus, TEM validates EV shape and contamination, BCA indicates elevated protein content, SDS-PAGE discloses varied protein profiles of EVs, and NTA offers detailed size and concentration metrics of crude and purified Foc-EVs, demonstrating that these methodologies effectively ascertain EV yield and purity in the absence of particular biomarkers.

### 4.4. Crude EVs Exhibit Enhanced Pathogenicity Due to Both EV and Non-EV-Containing Proteins

Our pathogenicity studies showed that crude EVs caused the most significant lesions on banana leaves, averaging 2.805 cm^2^, while Fraction 2 and Fraction 3 produced mild lesion areas of 1.386 cm^2^ and 1.255 cm^2^, respectively (Figure 3A,B). Similarly, EVs from the cotton pathogen *F. oxysporum* f. sp. *vasinfectum* (Fov) and the citrus pathogen *Penicillium digitatum* have phytotoxic effects on their hosts. *P. digitatum* EVs induced color alteration and tissue lesion formation in the *C. sinensis* seeds around the infiltration sites [13]. After five days, the Fov EVs prompted the development of necrosis on the cotyledon leaf at the infiltration site, indicating phytotoxicity [10]. When EVs were isolated from Fov, a unique purple color feature of the Fov-EVs was observed, which was predicted to arise from a naphthoquinone pigment [10].

In addition to the pathogenicity impact, we also demonstrated the biocontrol effect of gradient and crude *Foc* TR4-EVs on its spore germination (Figure 4A). We found that TR4-EVs have no significant effect on spore germination (Figure 4B). The lack of effect of *Foc* TR4-EVs on spore germination could be due to the specific nature of the EV cargo, which may not include components that directly inhibit or promote germination. Additionally, it is possible that the EVs’ impact is more pronounced at later stages of infection but needs to be further addressed. Correspondingly, the biocontrol capability of *A. pullulans* EVs on the spore germination of *Colletotrichum acutatum*, *Botrytis cinerea*, and *Penicillium expansum* was previously analyzed on the PDA plates. They found no reduction in spore germination for any of the tested phytopathogenic fungi after five days of incubation at 24 °C. All phytopathogenic fungi had the same colony diameter regardless of whether the spores were mixed with EVs or with sterile DPBS as a control prior to inoculation [29].

### 4.5. Fraction 2 Shows EV-Specific Functions, While Crude EVs Reflect Non-EV Unique Proteins

The functional capabilities of EVs are directly linked to their protein composition [46]. Our comparison focused on Fraction 2 and crude EVs, while the proteomic profile of Fraction 3 needs to be explored further. Proteomic analysis of purified EVs (Fraction 2, F2) and crude EVs identified 1399 and 771 total proteins, highlighting significant differences in their composition (Figure 5A). The number of proteins in purified and crude EVs varies, consistent with recent studies of purified EVs from plant pathogens like *H. capsulatum* 1410 proteins [47] and fungal EVs that are extracted without gradient, like *Z. tritici* 240 proteins [14].

The functional classification highlights typical proteins in Foc-EVs, such as transport, metabolism, signaling, pathogenic, cell wall and membrane integrity, biomarker, and vesicular trafficking (Figure 5B). Similar functional categories are also reported in *Botrytis cinereal, H. capsulatum*, and *A. infectoria* EVs, which contain proteins involved in polysaccharide metabolism, cell wall membrane integrity, and vesicle trafficking [47,48,49]. Further, proteasome, endocytosis, lysosome, and phagosome-related processes in F2 EVs contribute to their increased pathogenicity effect on banana leaves [50,51]. Proteomic analysis of EVs from *F. graminearum*, *P.capsica*, *F. vasinfectum*, *B.cinerea*, and *Z. tritici* have identified proteins and effectors linked to proteasome and endocytosis plant pathogenicity [10,12,14,48,52]. Furthermore, Fraction 2 also carried SNARE interactions, ABC transporters, MAPK, and RAP1 signaling pathways (Figure 5B). Recently, SNARE proteins were discovered as fungal EV biomarkers in *C. higginsianum*, a leaf spot disease pathogen [9]. These SNARE proteins are abundantly present in F2 unique and common EV proteins while absent in crude EV unique proteins.

Conversely, crude EVs exhibited thick protein bands on gels, suggesting higher protein content of EV proteins and also due to non-EV proteins co-purifying during isolation, revealed by KEGG and GO analysis (Figure 3 and Figure 5A,B). We propose that non-EV proteins related to oxidative phosphorylation, secondary metabolites, and metabolic pathways may present higher concentrations in Fraction 3, potentially contributing to the necrosis observed in banana leaves (Figure 3). However, further exploration of Fraction 3 through proteomic analysis is needed to confirm this. The higher molecular weight bands on gels reflect the abundance of these proteins. The lower number of proteins during proteomics in crude EVs is also due to these higher abundance proteins. These proteins can mask the detection of lower molecular weight EV proteins, resulting in a reduced number of identified proteins [53,54,55]. High-abundance contaminants can overshadow lower-abundance EV proteins [55]. This masking effect explains that certain proteins detected in Fraction 2 are not observed in the crude EV sample. Crude TR4-EVs displayed a higher abundance of metabolism-related proteins (36), suggesting that the crude preparation may be enriched with metabolic enzymes that mask the detection of lower molecular weight proteins. Therefore, the proteins observed exclusively in Fraction 2 are not unique to this fraction but are made more detectable during LC-MS/MS due to the reduction in contaminants during the purification process. Further, oxidative phosphorylation, phagosome, and endocytosis are possible pathways that cause necrosis in banana leaves [51].

The name and number of genes in pure and crude TR4-EVs vary significantly, especially in pathways linked to endocytosis, transportation, metabolism, pathogenicity, and signaling (Table 1). The differentially enriched proteins (DEPs) analysis suggests a balanced protein regulation in upregulated and downregulated proteins between F2 and crude EVs; however, the functional mechanism of these proteins differs significantly, as revealed by GO and KEGG (Figure 5D, Table 1). These findings highlight the value of purifying methods as they provide a more realistic representation of EV-specific proteins with extracellular functions.

## 5. Conclusions

**Figure 6:**
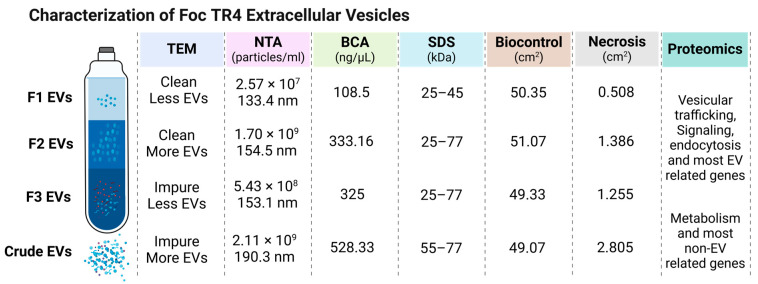
Comparative characterization of *Foc* TR4 EVs across four types: F1, F2, F3, and crude EVs. F2 EVs emerge as a promising candidate due to their high particle count, excellent purity, elevated protein levels, and specific enrichment in vesicular trafficking and signaling-related proteins, contributing to their pathogenicity on banana leaves. In contrast, crude EVs exhibit a higher particle count of larger-sized particles with cellular debris and contain high molecular weight proteins related to metabolism and secondary metabolites, which contribute to the highest necrosis observed on banana leaves.

This study demonstrated that techniques such as TEM, NTA, BCA, and SDS-PAGE effectively confirmed the isolation of Foc-EVs without relying on specific biomarkers. Further, both crude and purified EVs from *Foc* TR4-EVs induce lesions on banana leaves. However, proteomic analysis indicates that lesions from purified EVs are caused by proteins that are unique to EVs, but lesions from crude EVs also arose from non-EV proteins (Figure 6). The gradient and purification of EVs are crucial for accurately assessing the actual pathogenic effects of fungal EVs. Despite these insights, the current EV isolation methods have certain limitations, such as the potential for EV loss during gradient purification, highlighting the need for future studies to optimize techniques that improve yield and purity while minimizing sample loss. Hence, this study highlights the potential of targeting EV-associated proteins of Foc-TR4 as a novel strategy for banana disease management and controlling fungal virulence effectively.

## Figures and Tables

**Table 1:** Comparison of KEGG pathway genes and their predicted pathogenic roles identified in purified and crude *Foc* TR4-EVs.

Pathway	Genes #	Genes Names	Pathogenic Role
F2 TR4-EVs			
Endocytosis	24	*FOIG_gene0000606*; *FOIG_gene00745*; *FOIG_gene00658*; *FOIG_gene00641*; *FOIG_gene00616*; *FOIG_gene00601*; *FOIG_gene00617*; *FOIG_gene00682*; *FOIG_gene00681*; *FOIG_gene00778*; *FOIG_gene00787*; *FOIG_gene00801*; *FOIG_gene00867*; *FOIG_gene00977*; *FOIG_gene01014*; *FOIG_gene01020*; *FOIG_gene01027*; *FOIG_gene01081*; *FOIG_gene01084*; *FOIG_gene01101*; *FOIG_gene01191*; *FOIG_gene01235*; *FOIG_gene01285*; *FOIG_gene01304*	Host cell entry; intracellular trafficking
ABC transporters	12	*FOIG_gene00710*; *FOIG_gene00777*; *FOIG_gene00784*; *FOIG_gene00805*; *FOIG_gene00818*; *FOIG_gene00954*; *FOIG_gene01076*; *FOIG_gene01178*; *FOIG_gene01229*; *FOIG_gene01247*; *FOIG_gene01280*; *FOIG_gene01376*	Transport virulence factors; toxins; nutrients
Vesicular transport	6	*FOIG_gene00650*; *FOIG_gene00906*; *FOIG_gene00947*; *FOIG_gene01082*; *FOIG_gene01085*; *FOIG_gene01193*	Transport of virulence factors; facilitate infection
MAPK signaling	24	*FOIG_gene00699*; *FOIG_gene00709*; *FOIG_gene00674*; *FOIG_gene00755*; *FOIG_gene00640*; *FOIG_gene00681*; *FOIG_gene00758*; *FOIG_gene00785*; *FOIG_gene00789*; *FOIG_gene00866*; *FOIG_gene00919*; *FOIG_gene00968*; *FOIG_gene00978*; *FOIG_gene00991*; *FOIG_gene01057*; *FOIG_gene01072*; *FOIG_gene01089*; *FOIG_gene01102*; *FOIG_gene01109*; *FOIG_gene01113*; *FOIG_gene01145*; *FOIG_gene01249*; *FOIG_gene01361*; *FOIG_gene01388*	Stress adoptation; virulence regulation
Autophagy—yeast	23	*FOIG_gene00621*; *FOIG_gene00658*; *FOIG_gene00723*; *FOIG_gene00701*; *FOIG_gene00597*; *FOIG_gene00601*; *FOIG_gene00682*; *FOIG_gene00808*; *FOIG_gene00825*; *FOIG_gene00831*; *FOIG_gene00882*; *FOIG_gene00924*; *FOIG_gene00947*; *FOIG_gene01040*; *FOIG_gene01084*; *FOIG_gene01085*; *FOIG_gene01094*; *FOIG_gene01145*; *FOIG_gene01200*; *FOIG_gene01248*; *FOIG_gene01257*; *FOIG_gene01258*; *FOIG_gene01373*	Cellular survival; immune evasion
Proteasome	14	*FOIG_gene00644*; *FOIG_gene00740*; *FOIG_gene00661*; *FOIG_gene00598*; *FOIG_gene00680*; *FOIG_gene00835*; *FOIG_gene00890*; *FOIG_gene00958*; *FOIG_gene00990*; *FOIG_gene01063*; *FOIG_gene01087*; *FOIG_gene01186*; *FOIG_gene01231*; *FOIG_gene01341*	Protein degradation; iImmune modulation
Lipid metabolism	10	*FOIG_gene00737*; *FOIG_gene00611*; *FOIG_gene00626*; *FOIG_gene00713*; *FOIG_gene01043*; *FOIG_gene00773*; *FOIG_gene01048*; *FOIG_gene01227*; *FOIG_gene01361*; *FOIG_gene01370*	Membrane composition modulation
Common TR4-EVs			
Endocytosis	21	*FOIG_gene0046*; *FOIG_gene0072*; *FOIG_gene0073*; *FOIG_gene0084*; *FOIG_gene00119*; *FOIG_gene00122*; *FOIG_gene00150*; *FOIG_gene00172*; *FOIG_gene00202*; *FOIG_gene00218*; *FOIG_gene00221*; *FOIG_gene00222*; *FOIG_gene00234*; *FOIG_gene00243*; *FOIG_gene00245*; *FOIG_gene00259*; *FOIG_gene00352*; *FOIG_gene00379*; *FOIG_gene00388*; *FOIG_gene00463*; *FOIG_gene00475*	Host colonization; manipulation of host cell machinery
ABC transporters	7	*FOIG_gene0031*; *FOIG_gene0036*; *FOIG_gene0089*; *FOIG_gene00100*; *FOIG_gene00238*; *FOIG_gene00458*; *FOIG_gene00507*	Transportation and fungal adaptation
Vesicular transport	4	*FOIG_gene0085*; *FOIG_gene00120*; *FOIG_gene00332*; *FOIG_gene00467*	Virulence factors secretion
MAPK signaling	17	*FOIG_gene0028*; *FOIG_gene0075*; *FOIG_gene00156*; *FOIG_gene00202*; *FOIG_gene00207*; *FOIG_gene00221*; *FOIG_gene00245*; *FOIG_gene00250*; *FOIG_gene00273*; *FOIG_gene00335*; *FOIG_gene00352*; *FOIG_gene00389*; *FOIG_gene00412*; *FOIG_gene00471*; *FOIG_gene00504*; *FOIG_gene00523*; *FOIG_gene00544*	Virulence factor production; stress response; host immune defense
Autophagy—yeast	12	*FOIG_gene006*; *FOIG_gene00150*; *FOIG_gene00172*; *FOIG_gene00272*; *FOIG_gene00274*; *FOIG_gene00343*; *FOIG_gene00416*; *FOIG_gene00453*; *FOIG_gene00467*; *FOIG_gene00470*; *FOIG_gene00472*; *FOIG_gene00475*	Cellular homeostasis and pathogen survival under stress
Proteasome	3	*FOIG_gene0033*; *FOIG_gene00167*; *FOIG_gene00375*	Degradation of host immune factors
Glycerophospholipid metabolism	18	*FOIG_gene0022*; *FOIG_gene0061*; *FOIG_gene0071*; *FOIG_gene0078*; *FOIG_gene0087*; *FOIG_gene00114*; *FOIG_gene00141*; *FOIG_gene00152*; *FOIG_gene00159*; *FOIG_gene00217*; *FOIG_gene00280*; *FOIG_gene00325*; *FOIG_gene00339*; *FOIG_gene00358*; *FOIG_gene00405*; *FOIG_gene00409*; *FOIG_gene00579*; *FOIG_gene00580*	Membrane flexibility; fungal adaptability
Crude TR4-EVs			
Endocytosis	2	*FOIG_gene01418*; *FOIG_gene01495*	Establishment of infection
ABC transporters	6	*FOIG_gene01568*; *FOIG_gene01402*; *FOIG_gene01537*; *FOIG_gene01459*; *FOIG_gene01537*; *FOIG_gene01568*	Resistance to antifungal compounds
Vesicular transport	0	*FOIG_gene00650*; *FOIG_gene00906*; *FOIG_gene00947*; *FOIG_gene01082*; *FOIG_gene01085*; *FOIG_gene01193*	Secretion of effector proteins
MAPK signaling	2	*FOIG_gene01499*; *FOIG_gene01569*	Survival; pathogenesis
Autophagy—yeast	1	*FOIG_gene01499*	Cellular integrity
Proteasome	5	*FOIG_gene01405*; *FOIG_gene01411*; *FOIG_gene01452*; *FOIG_gene01470*; *FOIGgene01505*	Degradation of host proteins
Metabolism	36	*FOIG_gene01426*; *FOIG_gene01400*; *FOIG_gene01417*; *FOIG_gene01404*; *FOIG_gene01416*; *FOIG_gene01409*; *FOIG_gene01429*; *FOIG_gene01432*; *FOIG_gene01437*; *FOIG_gene01450*; *FOIG_gene01451*; *FOIG_gene01470*; *FOIG_gene01478*; *FOIG_gene01480*; *FOIG_gene01487*; *FOIG_gene01491*; *FOIG_gene01492*; *FOIG_gene01493*; *FOIG_gene01500*; *FOIG_gene01527*; *FOIG_gene01532*; *FOIG_gene01535*; *FOIG_gene01542*; *FOIG_gene01543*; *FOIG_gene01545*; *FOIG_gene01546*; *FOIG_gene01548*; *FOIG_gene01554*; *FOIG_gene01559*; *FOIG_gene01561*; *FOIG_gene01562*; *FOIG_gene01563*; *FOIG_gene01564*; *FOIG_gene01566*; *FOIG_gene01570*; *FOIG_gene01572*	Fungal growth; survival

## Data Availability

The datasets generated and/or analyzed during the current study, including proteomics data and associated protein accession numbers, are available from the corresponding author upon reasonable request.

## Supplementary Materials

The following supporting information can be downloaded at https://www.mdpi.com/article/10.3390/plants13243534/s1; Figure S1: Isolation and gradient purification of *Foc* TR4-EVs using the ultracentrifugation method; Table S1: Quantitative Real-Time PCR (qPCR) primers used in this study.
