# Supplementary figures and images for: Isolation, Characterization, and Proteomic Analysis of Crude and Purified Extracellular Vesicles Extracted from Fusarium oxysporum f. sp. cubense
Source: Plants (Basel). 2024 Dec 18;13(24):3534. doi: 10.3390/plants13243534 (PMC11679526; doi:10.3390/plants13243534)

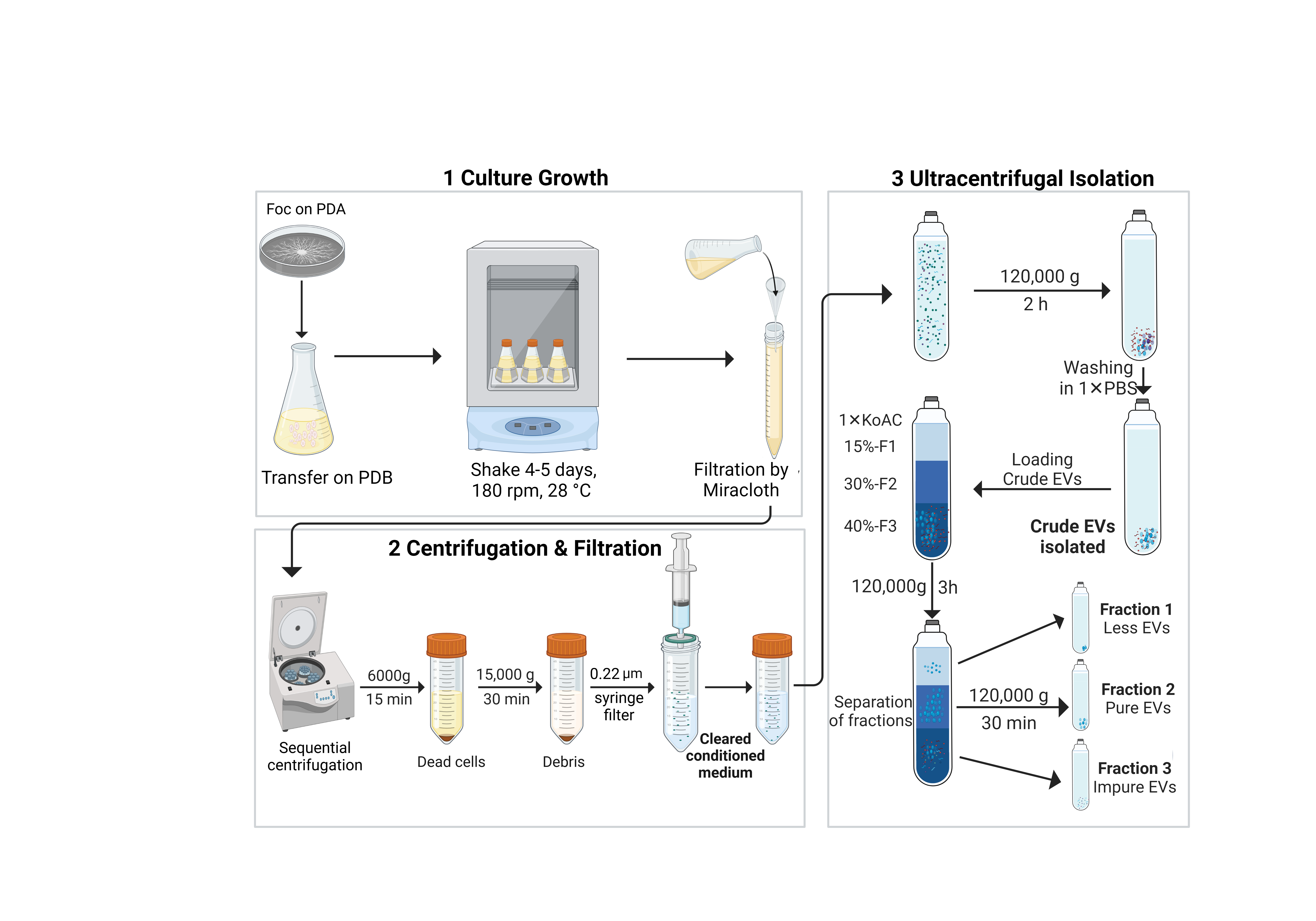

Supplement: Supplementary file 1 [file plants-13-03534-s001.zip › Figure S1.png]
